# Supplementary material for: Proteomic compensation by paralogs preserves protein interaction networks after gene loss in cancer
Source: Mol Syst Biol. 2025 May 28;21(8):8. doi: 10.1038/s44320-025-00122-4 (PMC12322171; doi:10.1038/s44320-025-00122-4)
Supplement: Supplementary file 1 — Appendix [file 44320_2025_122_MOESM1_ESM.pdf]

Appendix

Title: Paralog protein compensation preserves protein-protein interaction networks following gene loss in cancer

Authors:

Anjan Venkatesh[1,2,3], Niall Quinn[2,3], Swathi Ramachandra Upadhya[1,3], Barbara De Kegel[2,3], Alfonso Bolado Carrancio[4], Thomas Lefeivre[1,2,3], Olivier Dennler[1,3], Kieran Wynne[1,2,5], Alexander von Kriegsheim[4], Colm J. Ryan[1,2,3,5 *]

1. Conway Institute of Biomolecular and Biomedical Research, University College Dublin, Dublin, Ireland

2. Systems Biology Ireland, University College Dublin, Dublin, Ireland

3. School of Computer Science, University College Dublin, Dublin, Ireland

4. Edinburgh Cancer Research UK Centre, University of Edinburgh, Edinburgh, UK

5. School of Medicine, University College Dublin, Dublin, Ireland

* Corresponding author (Colm J. Ryan, colm.ryan@ucd.ie)

Table of Contents

Item	Page number
Appendix Figure S1	2
Appendix Figure S2	3
Appendix Figure S3	4-5
Appendix Figure S4	6
Appendix Figure S5	7
Appendix Figure S6	8
Appendix Figure S7	9
Appendix Figure S8	10
Appendix Figure S9	11
Appendix Figure S10	12

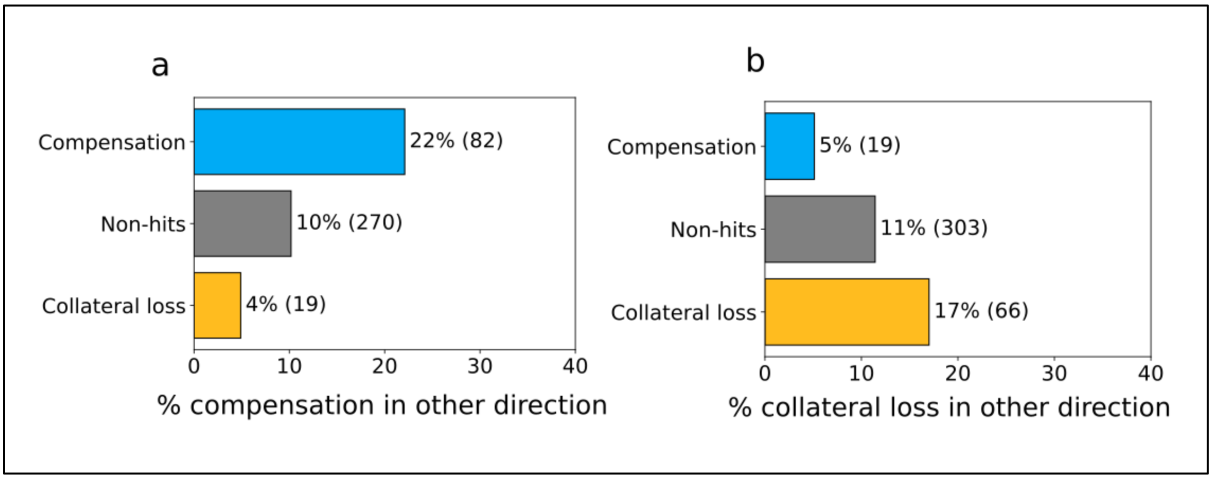

Appendix Figure S1. Paralog pairs tested in both directions are enriched for reciprocal hits (a) Bar chart showing the percentage of pairs in the compensation, non-hit, and collateral loss groups that are compensation hits in the other direction. **(b)** Similar bar chart showing the percentage of pairs in each set that are collateral loss hits in the other direction. Only pairs tested in both directions are considered here.

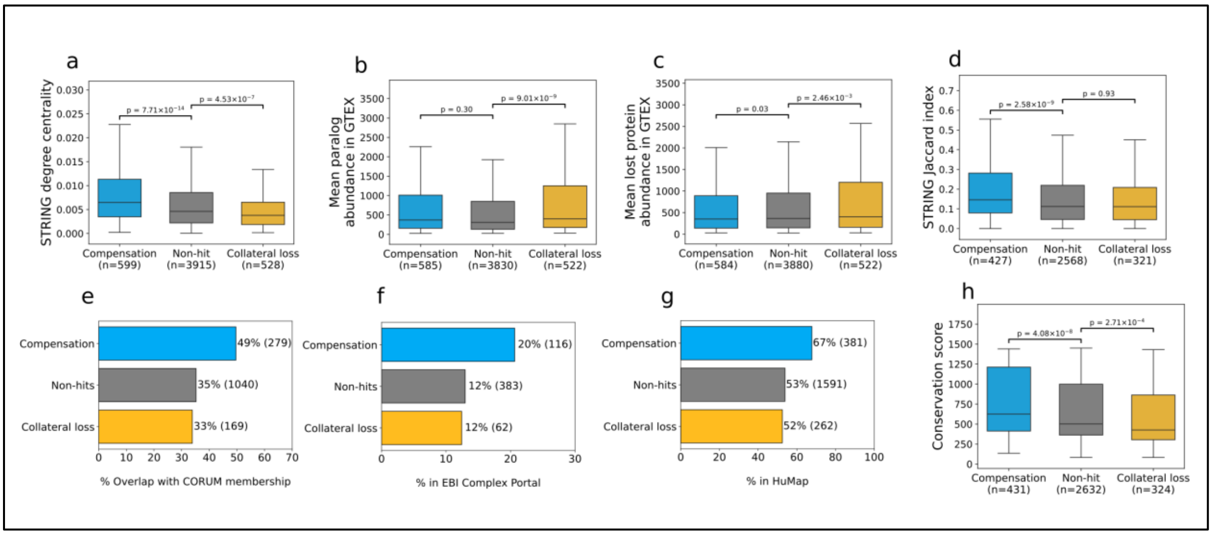

Appendix Figure S2. Compensation pairs are more central in the protein-protein interaction network when analyzing the STRING physical subnetwork, EBI ComplexPortal, and the HuMap database. (a) Box plot showing distributions of STRING degree centrality for compensation pairs, non-hits, and collateral loss pairs (Methods). **(b)** Box plot showing the mean abundance of the paralogous (not-lost) protein in the GTEx dataset of proteomic profiles from healthy tissues for all three groups. **(c)** Similar box plot showing the mean abundance of the lost protein in the GTEx dataset of proteomic profiles from healthy tissues for all three groups. **(d)** Similar box plot comparing the distributions of Jaccard indices calculated with the STRING network. **(e)** Bar chart showing the proportion of paralog pairs in each group where at least one paralog is a CORUM protein complex member (Methods). **(f)** Bar chart comparing the percentage of pairs in each set where either member of the pair is a member of a protein complex in EBI Complex Portal (Methods). **(g)** Similar bar chart plotted to show the percentages of pairs where either member is in a predicted HuMap complex (Methods). **(h)** Boxplots showing the conservation scores of paralog pairs in all three groups, using the presence of a known ortholog in other species as a proxy for conservation across evolutionary time (Methods). In all boxplots, the central line represents the median, box limits indicate the 25th and 75th percentiles (first and third quartiles), whiskers extend to 1.5 × interquartile range from either end of the box, and outliers are not displayed. Sample sizes are shown in parentheses and all p-values shown correspond to two-sided equal variance t-tests.

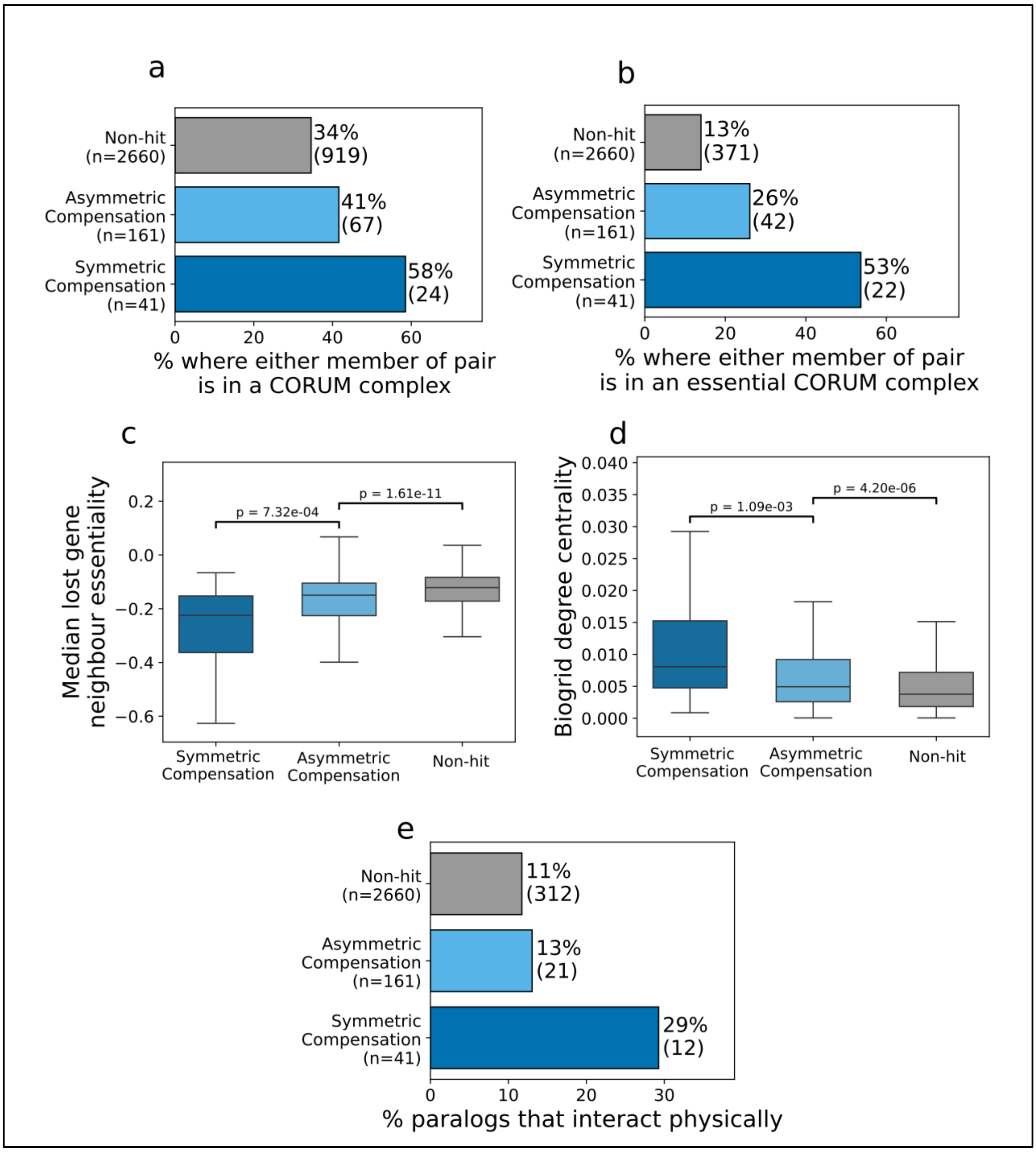

Appendix Figure S3. Symmetric compensation hits show greater enrichment for protein complex membership, and have higher Jaccard indices and degree centrality than asymmetric hits. (a) Bar chart showing the percentage of symmetric compensation pairs, asymmetric compensation pairs, and non-hits that where either member of the pair is in a CORUM complex. **(b)** Similar bar chart showing the percentage of pairs in each set where either member of the pair is in an essential CORUM complex **(c)** Boxplots showing the distribution of essentiality scores for the lost paralogs in symmetric compensation pairs,

asymmetric compensation pairs, and non-hits. **(d)** Similar boxplots showing the distributions of degree centralities for the three groups. **(e)** Bar chart showing the percentage of pairs in each of these three sets where the paralogs in the pair physically interact. In both boxplots, the central line represents the median, box limits indicate the 25th and 75th percentiles (first and third quartiles), whiskers extend to 1.5 × interquartile range from either end of the box, and outliers are not displayed. Sample sizes are shown in parentheses and all p-values shown correspond to two-sided equal variance t-tests.

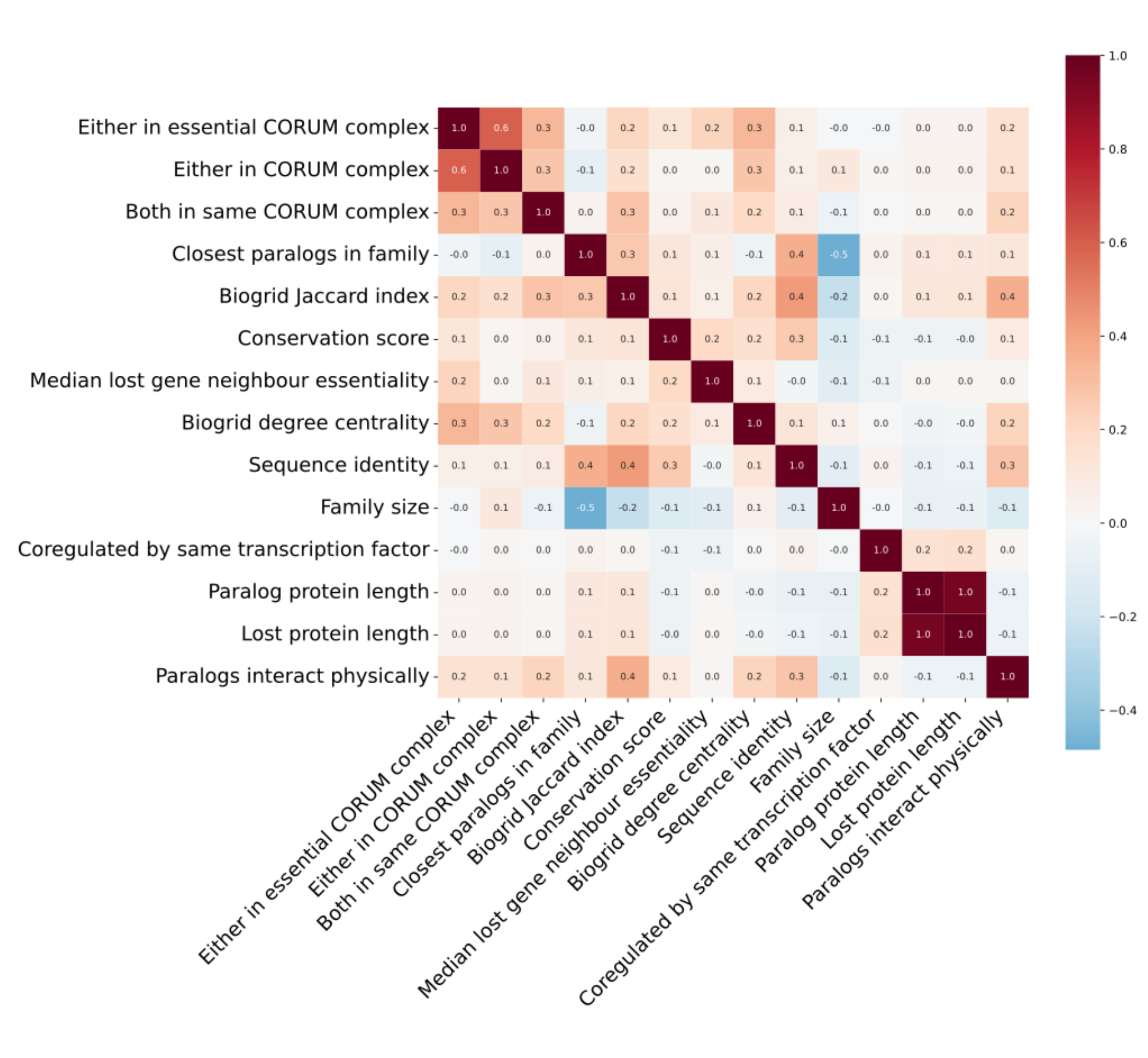

Appendix Figure S4. Most variables predictive of proteomic compensation are poorly correlated. Heatmap showing all-by-all Pearson correlations for variables assessed for the task of predicting proteomic compensation. Pearson correlation coefficients are displayed for each pairwise relationship.

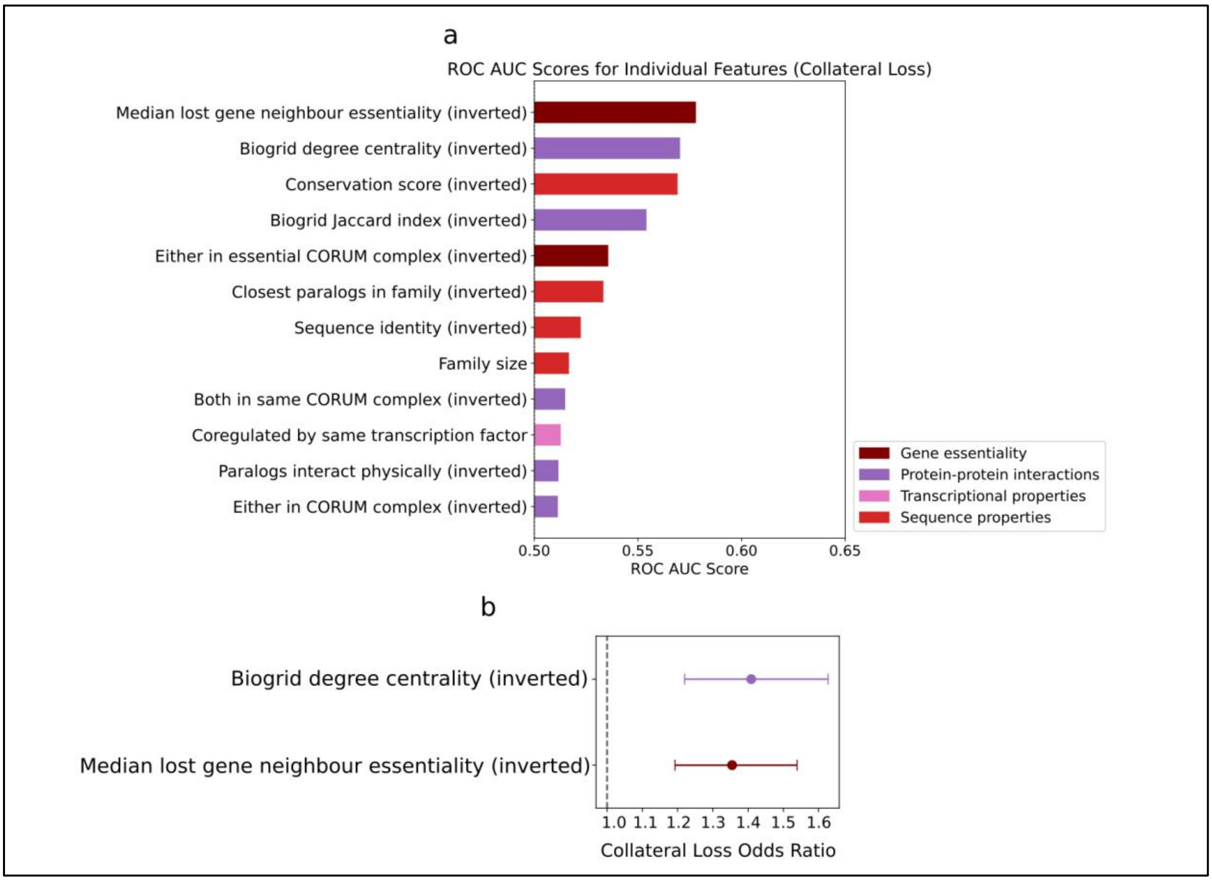

Appendix Figure S5. Collateral loss is predicted by degree centrality and neighbour essentiality. (a) Barchart showing ROC AUC values for 12 variables for predicting collateral loss (n = 3,863). **(b)** Dot plot showing odds ratios with error bars for individual variables in a LASSO regression model fit to predict collateral loss from the variables in (a).

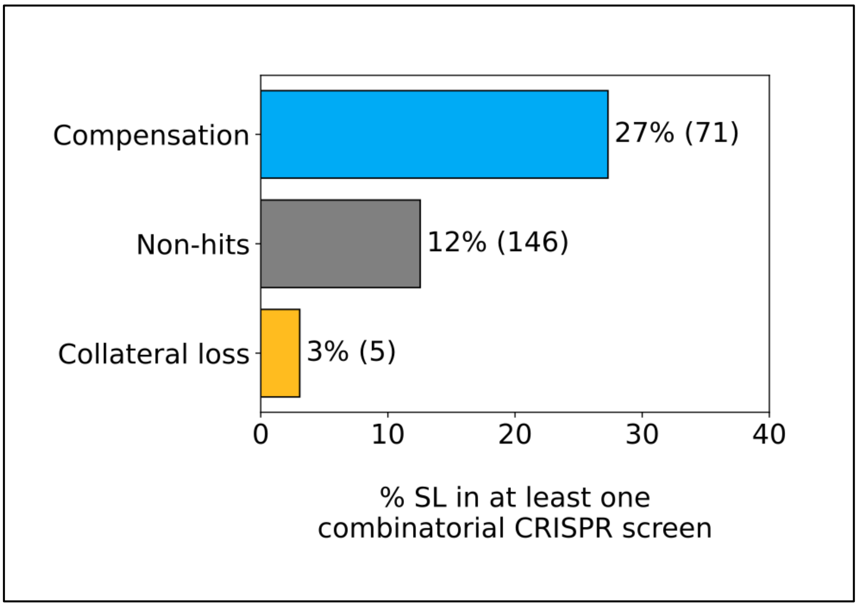

Appendix Figure S6. Compensation hits are enriched for paralog pairs that are synthetic lethal in at least one combinatorial CRISPR screen.

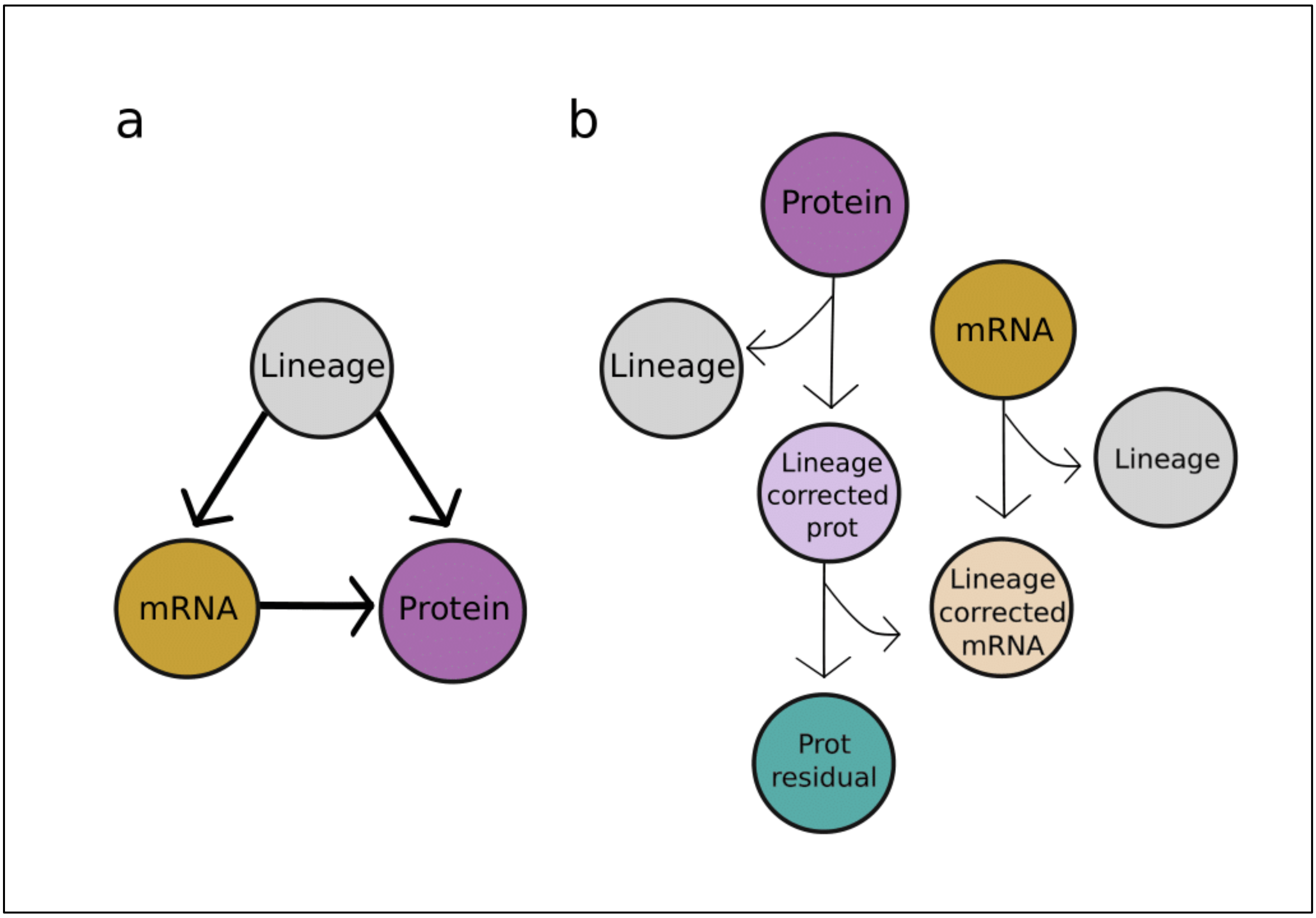

Appendix Figure S7. Generation of protein residual dataset by regressing out transcript abundance from protein abundance (a) Directed Acyclic Graph (DAG) showing the causal links between lineage/study, transcript abundance, and protein abundance. **(b)** Workflow diagram showing how mRNA abundance is regressed out from protein abundance while taking into account the effect of lineage on both variables.

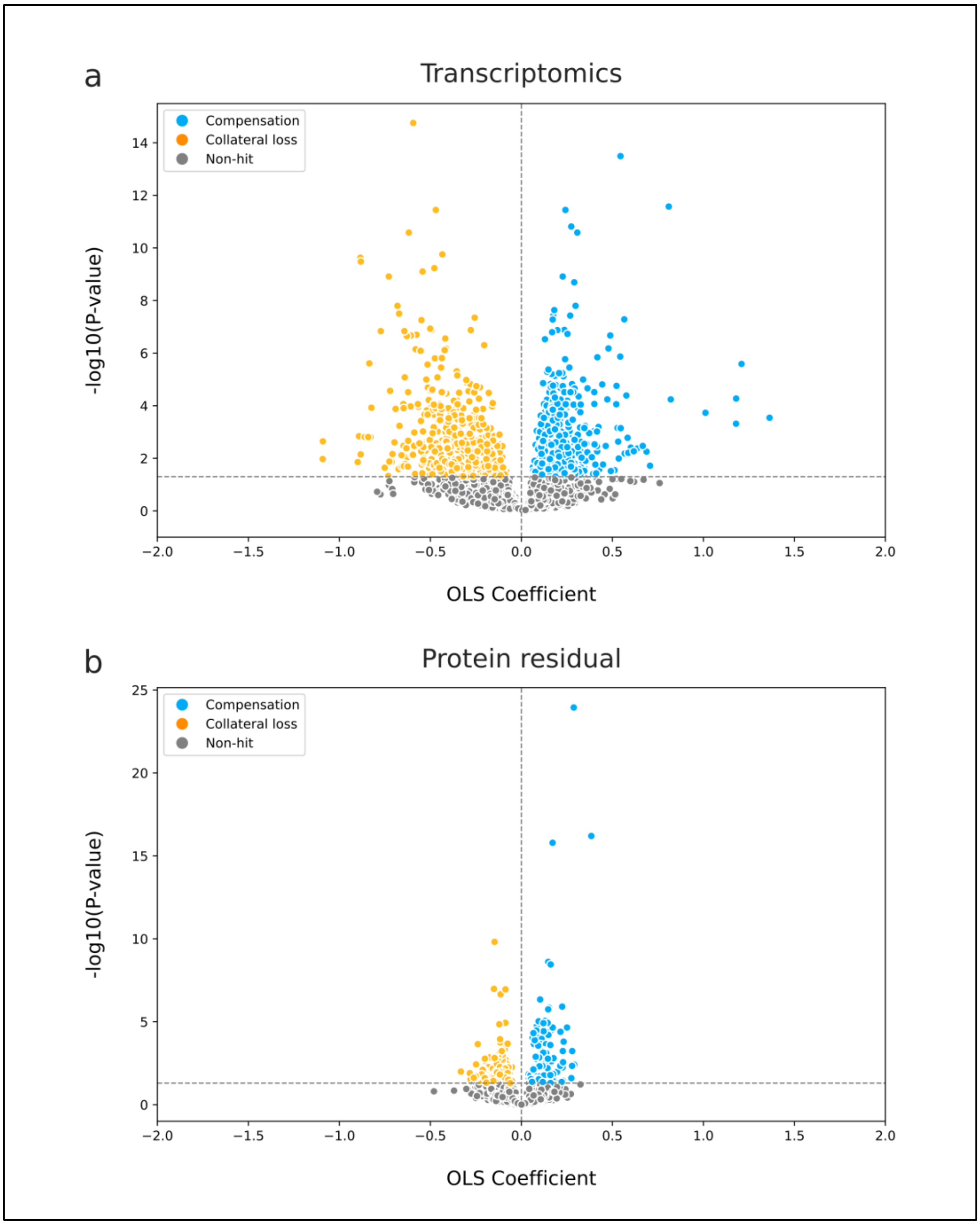

Appendix Figure S8. Volcano plots showing results for all paralog tests run with the CPTAC transcriptomic and protein residual datasets. Volcano plots show -log10(FDR) vs ordinary least squares (OLS) coefficient for all pairs tested. The same 5,128 pairs tested in the proteomic analysis were tested with these datasets.

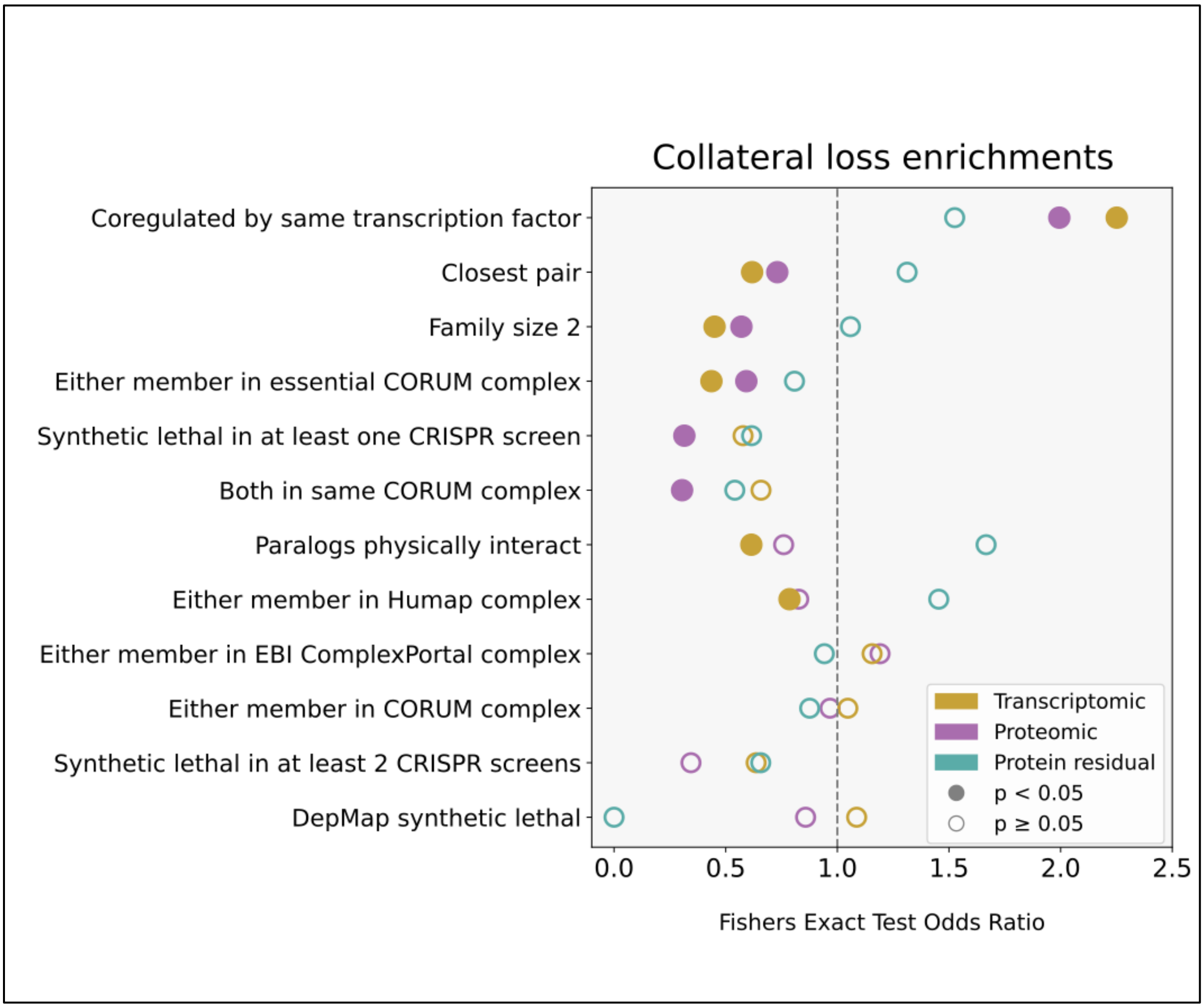

Appendix Figure S9. Collateral loss pairs are significantly less likely to be each other's closest paralog or only paralog and less likely to be members of protein complexes. Dot plot showing odds ratios of Fisher's Exact Tests checking enrichment for different variables among transcriptomic, proteomic, and protein residual collateral loss pairs.

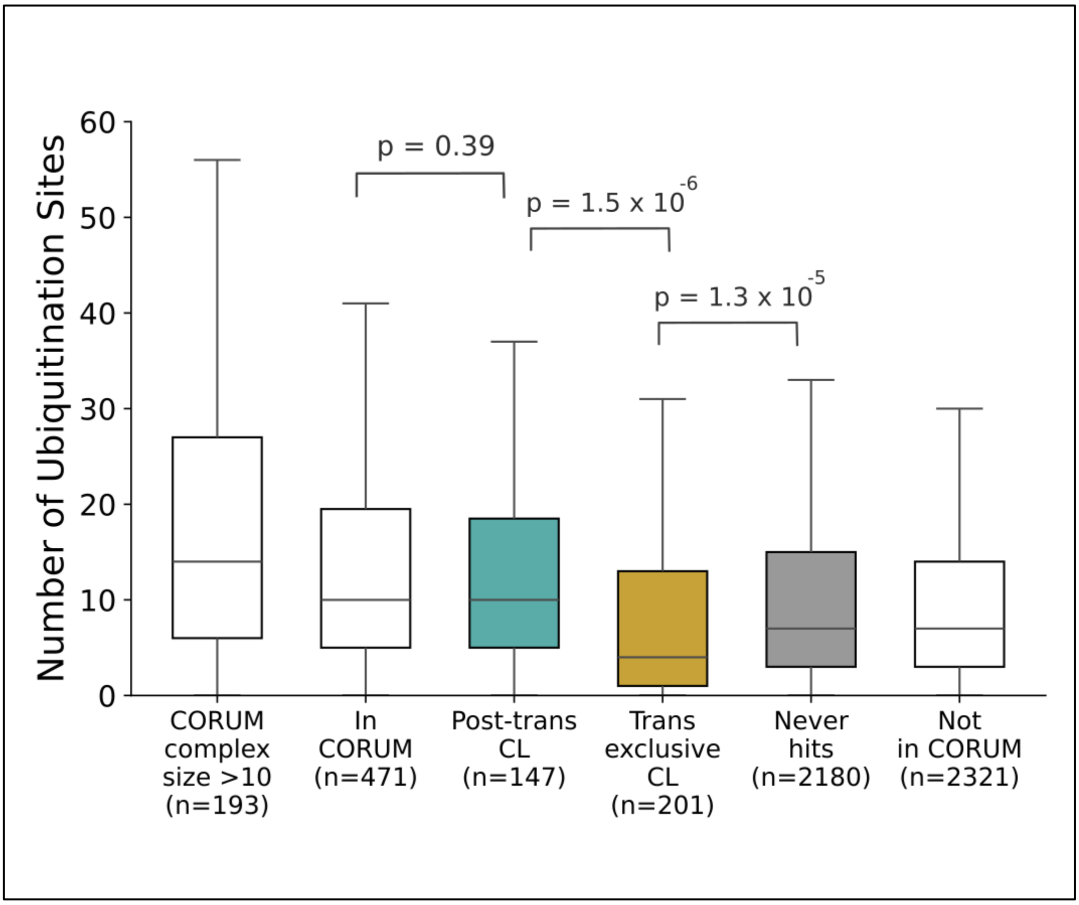

Appendix Figure S10. Post-transcriptional collateral loss pairs have significantly greater numbers of ubiquitination sites. Boxplots showing distributions of the numbers of ubiquitination sites in collaterally lost paralogs in pairs that are post-transcriptional (i.e. hits detected with the protein residual dataset but not the transcriptomic dataset) and transcriptional (i.e. hits detected with the transcriptomic dataset), compared to those in never-hit pairs, CORUM complex members, large CORUM complex members, and proteins that are never in a CORUM complex. The central line represents the median, box limits indicate the 25th and 75th percentiles (first and third quartiles), whiskers extend to 1.5 × interquartile range from either end of the box, and outliers are not displayed. Sample sizes are shown in parentheses and all p-values shown correspond to two-sided Mann-Whitney U tests.
