# Supplementary material for: Proteomic compensation by paralogs preserves protein interaction networks after gene loss in cancer
Source: Mol Syst Biol. 2025 May 28;21(8):8. doi: 10.1038/s44320-025-00122-4 (PMC12322171; doi:10.1038/s44320-025-00122-4)
Supplement: Supplementary file 13 — Expanded View Figures [file 44320_2025_122_MOESM13_ESM.pdf]

# Expanded View Figures

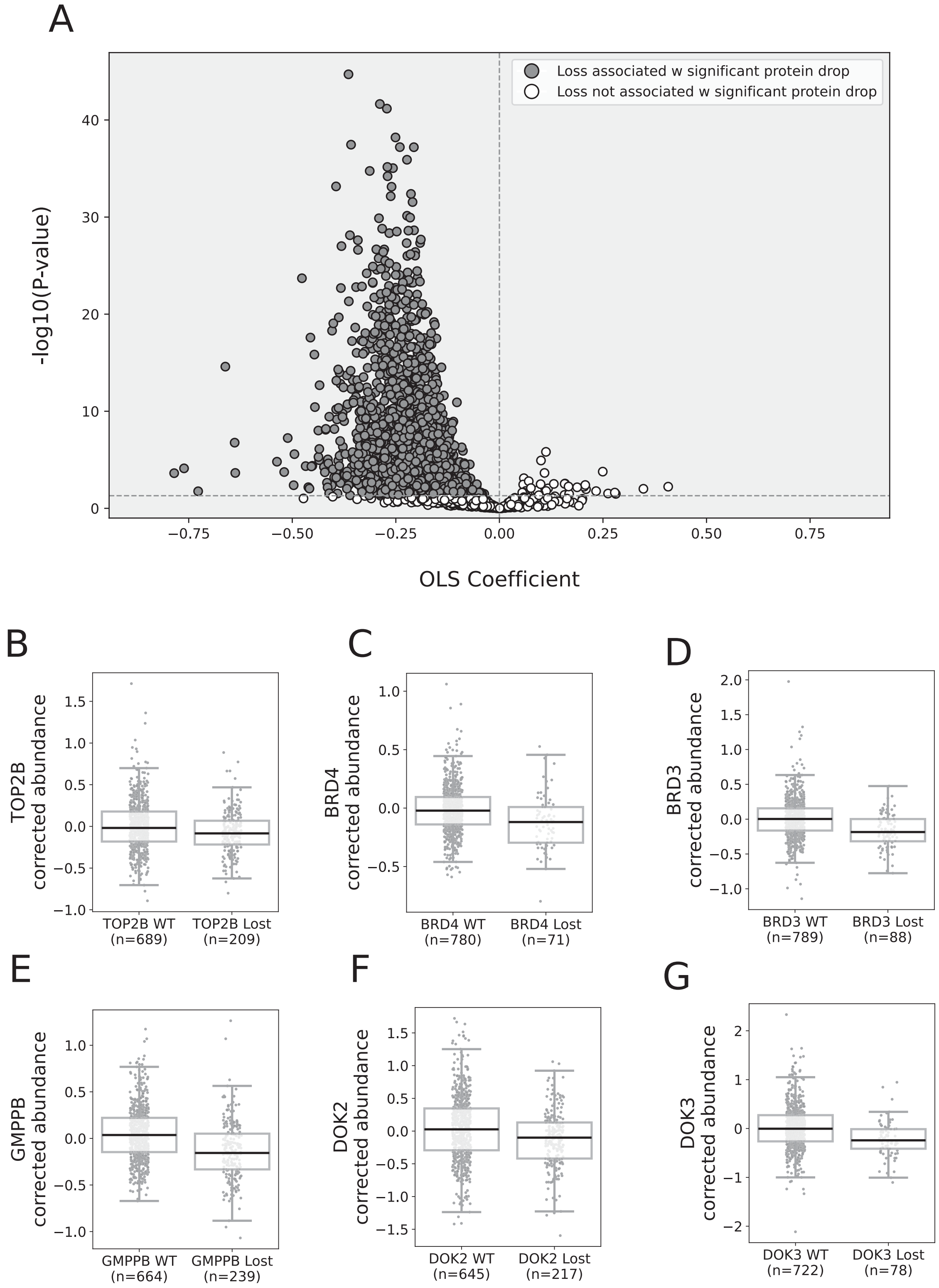


 © The Author(s)

◀ **Figure EV1. Most hemizygous losses are associated with a drop in protein abundance.**

(**A**) Volcano plot showing decrease in protein abundance of most tested proteins following hemizygous loss (out of 3439 tests run using CPTAC data) (**B–G**) Box plots comparing abundance of the 'lost' paralog in samples where it is hemizygously lost versus samples retaining both copies of the gene, for all pairs shown in Fig. 2 (*TOP2B*, *BRD4*, *BRD3*, *GMPPB*, *DOK2*, *DOK3*). In all boxplots, the central line represents the median, box limits indicate the 25th and 75th percentiles (first and third quartiles), and whiskers extend to 1.5 × interquartile range from either end of the box. Each grey dot represents a tumour sample. Position on the y-axis indicates protein abundance adjusted for copy number of the encoding gene and cancer type, which are used as covariates in the regression model. Sample sizes for each group are shown in parentheses. Sample sizes for each group are shown in parentheses.

© The Author(s)

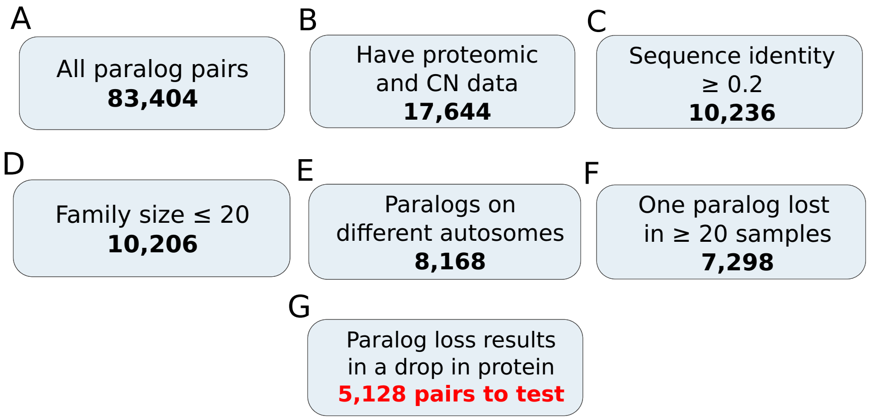


**Figure EV2. Workflow diagram showing the number of paralog pairs filtered out at each step.**

**A–G**. Number of paralog pairs remaining after applying each consecutive filtering step. 5128 paralog pairs are tested in the final analysis of CPTAC proteomic data.

 © The Author(s)

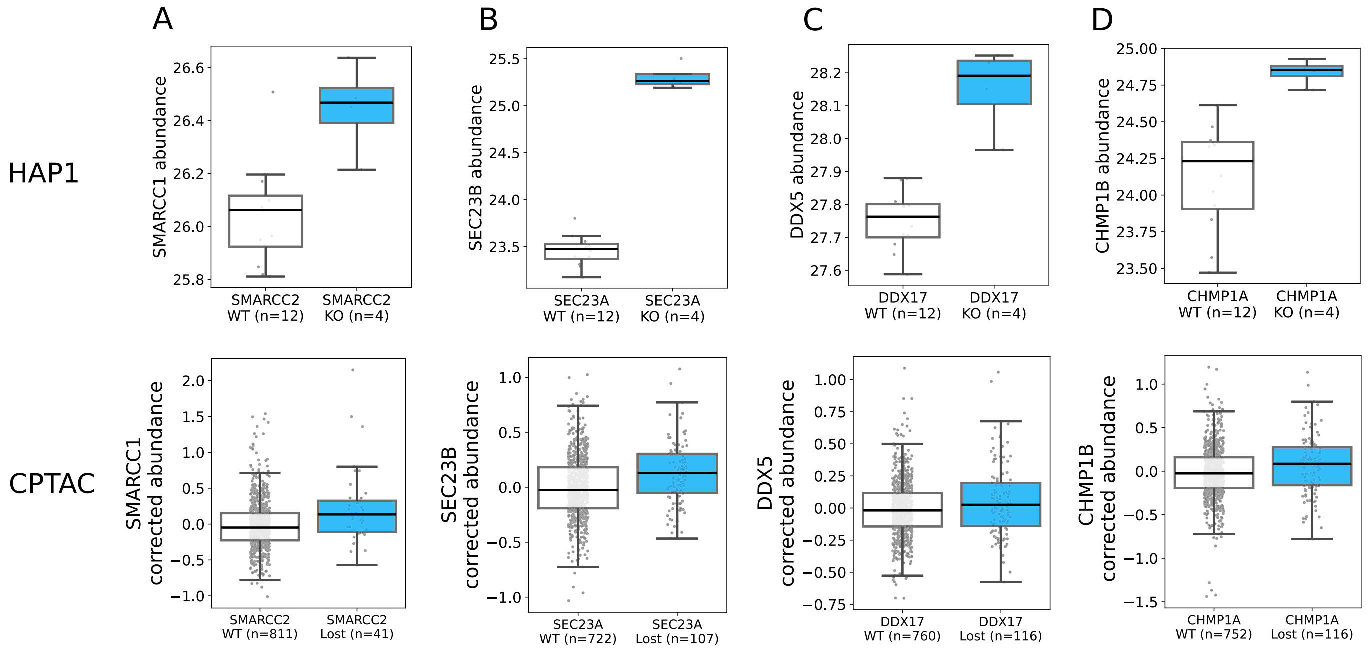


**Figure EV3. Four paralog pairs are compensation hits both with the HAP1 cell line dataset and the CPTAC tumour sample dataset.**

(**A**) Box plots showing increase in SMARCC1 protein abundance associated with *SMARCC2* knockout in a HAP1 background and *SMARCC2* loss in tumour samples. (**B**) Similar plots showing SEC23B abundance increase associated with *SEC23A* loss. (**C**) Similar plots showing DDX5 abundance increase associated with *DDX17* loss. (**D**) Similar box plots showing CHMP1B abundance increase associated with *CHMP1A* loss. In all boxplots, the central line represents the median, box limits indicate the 25th and 75th percentiles (first and third quartiles), and whiskers extend to 1.5 × interquartile range from either end of the box. Each grey dot represents a cell line in the HAP1 plots and a tumour sample in the CPTAC plots.

© The Author(s) 

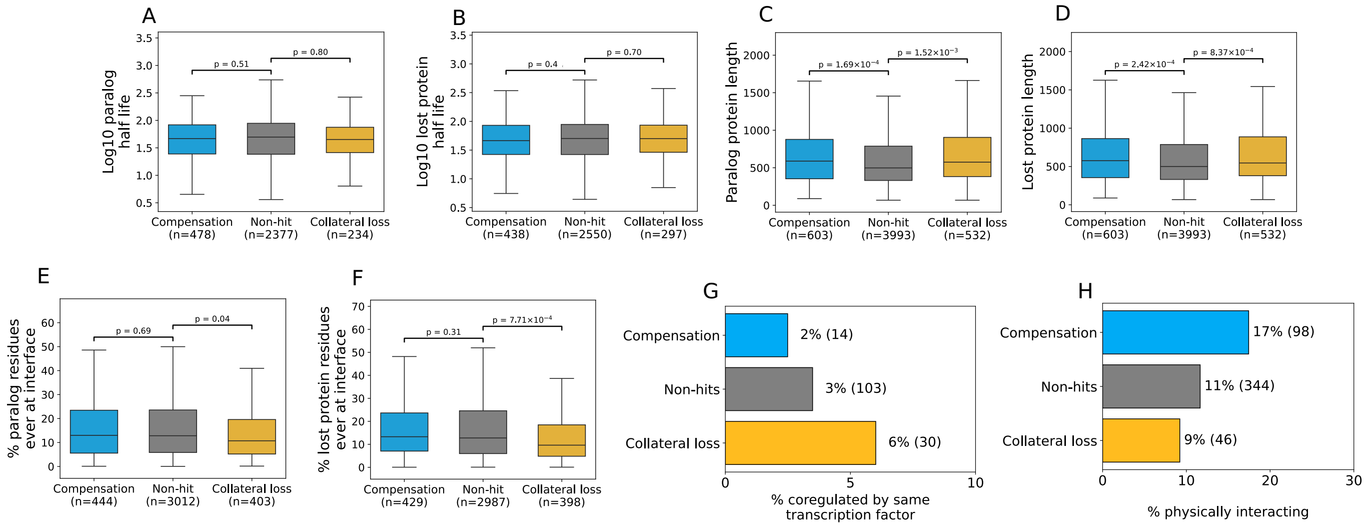


**Figure EV4. Paralogs in compensation and collateral loss pairs tend to be longer.**

Collateral loss paralogs tend to have fewer residues at protein–protein interaction interfaces and are more likely to be co-regulated by the same transcription factor. Compensation pairs are more likely to have a direct physical interaction. **(A)** Box plot showing the distributions of logged protein half-lives of paralogous proteins in the three groups. **(B)** Similar box plot showing the distributions of logged protein half-lives of the lost paralog in the three groups. **(C)** Box plot showing the distributions of protein lengths for paralogous (not lost) proteins in compensation pairs, non-hits, and collateral loss pairs. **(D)** Similar box plot showing the distributions of protein lengths for the lost paralog in the three groups. **(E)** Box plot showing the percentage of residues ever in a protein–protein interaction interface for paralogous proteins in compensation pairs, non-hits, and collateral loss pairs. **(F)** Similar box plot showing the percentage of residues ever in a protein–protein interaction interface for the lost paralogs in compensation pairs, non-hits, and collateral loss pairs. **(G)** Bar chart showing the percentage of compensation pairs, non-hits, and collateral loss pairs that are co-regulated by the same transcription factor. **(H)** Similar bar chart showing the percentage of pairs in the three groups that physically interact. In all boxplots, the central line represents the median, box limits indicate the 25th and 75th percentiles (first and third quartiles), whiskers extend to 1.5 × interquartile range from either end of the box, and outliers are not displayed. Sample sizes are shown in parentheses and all *p*-values shown correspond to two-sided equal variance t-tests.

 © The Author(s)

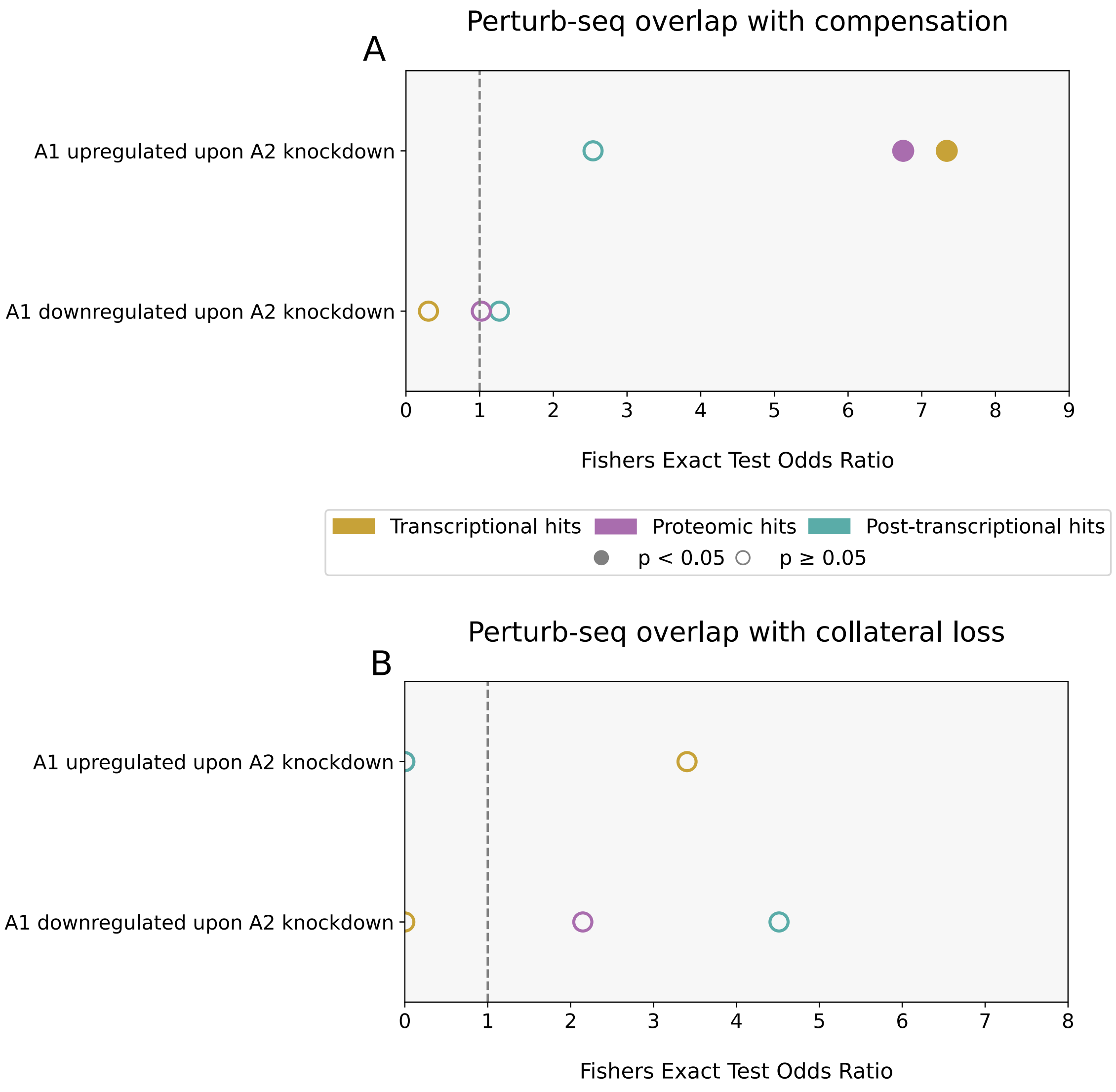


**Figure EV5. Only transcriptionally-driven compensation pairs are enriched for genes found to display upregulation upon paralog knockdown in a Perturb-seq experiment.**

(**A**) Dot plot showing odds ratios from Fisher's Exact tests assessing the enrichment of pairs found to be upregulated or downregulated upon paralog knockdown in a Perturb-seq experiment in three categories of pairs—transcriptional (compensation hits identified with the CPTAC transcriptomic dataset), post-transcriptional (compensation hits identified with the CPTAC protein residual dataset but not the transcriptomic dataset) and non-hits. (**B**) Similar dot plot showing enrichments for collateral loss hits in the three categories.

© The Author(s)
